# Haptoglobin and hemopexin inhibit vaso-occlusion and inflammation in murine sickle cell disease: Role of heme oxygenase-1 induction

**DOI:** 10.1371/journal.pone.0196455

**Published:** 2018-04-25

**Authors:** John D. Belcher, Chunsheng Chen, Julia Nguyen, Fuad Abdulla, Ping Zhang, Hao Nguyen, Phong Nguyen, Trevor Killeen, Sylvia M. Miescher, Nathan Brinkman, Karl A. Nath, Clifford J. Steer, Gregory M. Vercellotti

**Affiliations:** 1 Department of Medicine, Division of Hematology, Oncology and Transplantation, Vascular Biology Center, University of Minnesota, Minneapolis, Minnesota, United States of America; 2 CSL Behring AG, Research and Development, Bern, Switzerland; 3 CSL Behring, Research & Development, Kankakee, Illinois, United States of America; 4 Division of Nephrology and Hypertension, Mayo Clinic, Rochester, Minnesota, United States of America; 5 Department of Medicine, Division of Gastroenterology, Hepatology and Nutrition, University of Minnesota, Minneapolis, Minnesota, United States of America; Université Claude Bernard Lyon 1, FRANCE

## Abstract

During hemolysis, hemoglobin and heme released from red blood cells promote oxidative stress, inflammation and thrombosis. Plasma haptoglobin and hemopexin scavenge free hemoglobin and heme, respectively, but can be depleted in hemolytic states. Haptoglobin and hemopexin supplementation protect tissues, including the vasculature, liver and kidneys. It is widely assumed that these protective effects are due primarily to hemoglobin and heme clearance from the vasculature. However, this simple assumption does not account for the consequent cytoprotective adaptation seen in cells and organs. To further address the mechanism, we used a hyperhemolytic murine model (Townes-SS) of sickle cell disease to examine cellular responses to haptoglobin and hemopexin supplementation. A single infusion of haptoglobin or hemopexin (± equimolar hemoglobin) in SS-mice increased heme oxygenase-1 (HO-1) in the liver, kidney and skin several fold within 1 hour and decreased nuclear NF-ĸB phospho-p65, and vaso-occlusion for 48 hours after infusion. Plasma hemoglobin and heme levels were not significantly changed 1 hour after infusion of haptoglobin or hemopexin. Haptoglobin and hemopexin also inhibited hypoxia/reoxygenation and lipopolysaccharide-induced vaso-occlusion in SS-mice. Inhibition of HO-1 activity with tin protoporphyrin blocked the protections afforded by haptoglobin and hemopexin in SS-mice. The HO-1 reaction product carbon monoxide, fully restored the protection, in part by inhibiting Weibel-Palade body mobilization of P-selectin and von Willebrand factor to endothelial cell surfaces. Thus, the mechanism by which haptoglobin and hemopexin supplementation in hyperhemolytic SS-mice induces cytoprotective cellular responses is linked to increased HO-1 activity.

## Introduction

Polymerization of hemoglobin-S (HbS) in the deoxy conformation shortens the lifespan of sickle red blood cells (SS-RBCs) and promotes intravascular and extravascular hemolysis. When SS-RBCs are lysed intravascularly, HbS is released into the vascular space where it can consume nitric oxide and be oxidized to higher oxidative forms [1–3]. During these reactions, ferric (Fe^3+^) hemoglobin (metHb) is formed, which readily releases heme [4–6].

The released heme can activate the innate immune pattern recognition receptor toll-like receptor 4 (TLR4) on inflammatory cells, platelets and endothelium, promoting a pro-inflammatory and pro-coagulant phenotype, ultimately leading to vaso-occlusion, ischemia-reperfusion physiology, tissue injury, and pain in murine models of SCD [5, 7–10].

Haptoglobin and hemopexin are plasma proteins with the highest binding affinities for hemoglobin (Hb) (K_d_ = ~10^−12^ M) and heme (K_d_ < 10^−13^ M), respectively [11]. Haptoglobin and hemopexin render Hb and heme relatively nonreactive [12–16] and deliver Hb and heme safely to CD163 receptors on macrophages [17] and CD91 receptors on hepatocytes [18, 19], respectively, for endocytosis and degradation of their heme moieties by heme oxygenase-1 (HO-1) [19, 20]. We previously demonstrated that haptoglobin and hemopexin inhibit Hb- and heme-mediated microvascular stasis in SCD mice [5]. We hypothesized that haptoglobin and hemopexin prevented free heme from activating TLR4.

Plasma haptoglobin and hemopexin levels are often depleted in SCD patients and mice due to chronic intravascular hemolysis [21–24]. In animal models, increasing plasma haptoglobin or hemopexin can prevent organ toxicity caused byHb and heme [14, 22, 24–28]. Conversely, haptoglobin and hemopexin gene-null mice are especially prone to oxidative stress and inflammation [25, 26, 29–32].

To further explore mechanisms mediating haptoglobin and hemopexin inhibition of microvascular stasis and provide a basis for replacement therapy in SCD patients, we examined the role of HO-1 in haptoglobin and hemopexin-mediated protection.

## Materials and methods

### Reagents

Ferrous human hemoglobin (Hb) was purified from pooled human plasma under GLP conditions as described previously [33]. Endotoxin-free human albumin, haptoglobin and hemopexin were purified from pooled human plasma under GLP conditions and were supplied by CSL Behring (Bern, Switzerland). Lipopolysaccharide (LPS, *Escherichia coli* serotype O111:B4) was from Sigma-Aldrich.

### Mice

All animal experiments were approved by the University of Minnesota’s Institutional Animal Care and Use Committee. These studies utilized approximately equal numbers of male and female Townes-SS sickle mice on a 129/B6 mixed genetic background [34]. The SS mice were created by knocking in human α and ^A^γβ^S^ globins into the deletion sites for murine α- and β-globins. SS-mice have anemia and an SS-RBC half-life of 2.5 days (d). Townes-AA control mice express normal human α and ^A^γβ^A^ globins with a 16d RBC half-life [35]. All animals were housed in specific pathogen-free rooms to prevent infections on a 12 hour (h) light/dark cycle at 21°C. All animals were monitored daily including weekends and holidays for health problems, food and water levels and cage conditions. Littermates were randomly assigned to different treatment groups. No differences in endpoints were detected between male and female mice. All animals were included in each endpoint analysis and there were no unexpected adverse events that required modification of the protocol. Mice were aged 12.6 ± 2.5 weeks (mean ± SD) and weighed 24.0 ± 5.1 g (mean ± SD).

### Administration of hemoglobin, albumin, haptoglobin and hemopexin

All protein reagents were infused at equimolar heme and heme-binding capacity. Hb (1μmole heme/kg body weight) was infused via tail vein alone or in combination with vehicle (saline), albumin (1μmol/kg), haptoglobin (1μmol haptoglobin heme-binding capacity/kg), hemopexin (1μmol/kg), or haptoglobin+hemopexin (0.5μmol haptoglobin heme-binding capacity/kg + 0.5μmol hemopexin/kg). All infusion volumes were 10ml/kg body weight. In preliminary studies, the haptoglobin binding capacity (μmols heme/mg protein) was determined using various ratios of Hb:haptoglobin and size exclusion HPLC to measure Hb-haptoglobin complexes and free Hb. Hb was infused at the same time as albumin, haptoglobin and hemopexin. In some experiments haptoglobin and hemopexin were infused without Hb.

### Measurement of vaso-occlusion (microvascular stasis)

Townes-SS mice were implanted with dorsal skin-fold chambers (DSFCs) as previously described [36]. Three days later, mice with DSFCs were anesthetized with a mixture of ketamine (106mg/kg) and xylaxine (7.2mg/kg), placed on an intravital microscopy stage, and 20–24 flowing subcutaneous venules were selected and mapped. After baseline selection of venules, mice were infused with Hb, albumin, haptoglobin or hemopexin as described above. The same vessels were re-examined for stasis (no flow) at 1h and percent stasis was calculated. In other experiments, mice were pretreated with intravenous saline, haptoglobin, or hemopexin and then challenged with hypoxia-reoxygenation (H/R, 7% O_2_/93% N_2_ for 1h followed by room air for 1h) or LPS (1 mg/kg, i.p.).

### Administration of tin protoporphyrin (SnPP) and carbon monoxide (CO)

HO-1 activity was inhibited by administration of SnPP (40μmols/kg, i.p. X 3 days) with the third SnPP dose given before Hb, albumin, haptoglobin, hemopexin, H/R or LPS. In a second set of studies, mice were pretreated with SnPP and inhaled CO (250 ppm for 1h/day X 3 days) with the last dose of inhaled CO administered 1h prior to challenge with Hb.

### Collection of tissues

Plasma, livers, kidneys, and dorsal skin samples were collected from mice at the indicated time points after infusions. Heparinized blood was collected from the inferior vena cava, placed on ice, centrifuged at 500 X g. The collected plasma, liver and skin samples were flash-frozen in liquid N_2_ and stored at -85°C until use.

### Plasma hemoglobin and heme

Plasma Hb (ferrous Hb + metHb) was measured spectrophotometrically by the Winterbourn method [37]. Total plasma heme levels were measured colorimetrically at 400nm using a QuantiChrom^TM^ Heme Assay kit (BioAssay Systems). This method measures total plasma heme, including heme bound to protein.

### Immunoblots

Microsomes and nuclear extracts were isolated from tissues of mice as previously described [38]. Immunoblots of cellular subfractions were immunostained with primary antibodies to HO-1 (Enzo #ADI-OSA-111), NF-ĸB phospho-p65 (Ser536, Cell Signaling #3031) and total p65 (Cell Signaling #3034). Primary antibodies were labeled with the appropriate secondary antibodies conjugated to alkaline phosphatase (Santa Cruz, #SC-2007) and visualized with ECF™ substrate (GE Healthcare) and a Storm™ Reader (GE Healthcare). Immunoreactive bands on images were quantitated using ImageJ software (NIH). Mean relative expression of protein bands from each treatment group were calculated.

### Immunofluorescence staining for VCAM-1, ICAM-1, and CD31 in dorsal skin

For skin immunofluorescence staining, Townes-SS mice were infused with vehicle, Hb, Hb + haptoglobin, or Hb + hemopexin. After 4h, mice were sacrificed and dorsal skin samples were collected, fixed in phosphate buffered picric acid-formaldehyde fixative (Zamboni’s fixative), processed and cut into 100μm sections and analyzed as described [39] using primary antibodies to VCAM-1 (Bioss, #bs-0920R), ICAM-1 (Abcam, #ab124760), and CD31 (BD Pharmingen, #553370). Primary antibodies in tissues were identified with the appropriate fluorescent-labeled secondary antibodies. Slides were mounted using Vectashield™ antifade mounting medium with DAPI (Vector Laboratories), visualized, and images acquired using a FluoView FV1000 BX2 upright confocal microscope (Olympus, Center Valley, PA) with a 60X objective at room temperature and processed with FluoView (Olympus) and Adobe Photoshop software (San Jose, CA).

### Measurement of heme oxygenase enzyme activity in liver microsomes

Heme oxygenase (HO) activity was measured as previously described [40] in freshly isolated liver microsomes sonicated once for 10 seconds. Microsomes (2mg) in 2mM MgCl_2_, 0.1M K_2_HPO_4_ buffer, pH 7.4 were added to the reaction mixture (400μl, final) containing 2.5μg of recombinant biliverdin reductase (Assay Designs), 2mM glucose-6-phosphate, 0.2U glucose-6-phosphate dehydrogenase, 50μM hemin chloride (Frontier Scientific Porphyrin Products) and 0.8mM NADPH (Calbiochem) for 1h in the dark. Bilirubin that was formed was extracted into chloroform and measured by the delta O.D. at 464-530nm (extinction coefficient, 40mM^-1^ cm^-1^ for bilirubin). HO activity is expressed as pmol of bilirubin formed/mg microsomal protein/h.

### Immunofluorescence staining for P-selectin and von Willebrand factor (VWF) in human umbilical vein endothelial cells (HUVEC)

HUVEC were isolated from human umbilical cords and cultured as previously described [41]. HUVEC in 0.1% fetal bovine serum (FBS) were incubated at 37°C with media or media plus 80μM CO-releasing molecule (CORM) 1A or CORM 2 (Sigma Aldrich) for 30 minutes (m). After 30m, 10μM heme or 100μM histamine (positive control) was added for an additional 30m. Cells were fixed in 4% paraformaldehyde, stained with anti-P-selectin (R&D Systems) and anti-VWF IgG (Cedarlane). Cell surface bound antibody was visualized with appropriate fluorescent-labeled secondary IgG. Slides were mounted, visualized, and images acquired as described above for VCAM-1 and ICAM-1 immunofluorescence.

### Cytokines

Plasma chemokine ligand 5 (CCL5/RANTES), TNF-α, IL-10 and IFN-γ were measured by ELISA (Quansys Biosciences).

### 4-Hydroxynonenal (4-HNE)

4-HNE was measured in liver microsomes using an OxiSelect^TM^ HNE Adduct competitive ELISA kit (Cell Biolabs) according to the manufacturer’s instructions.

### Statistics

Analyses were performed with SigmaStat 3.5 for Windows (Systat Software, San Jose, CA). Comparisons of multiple treatment groups were made using one-way analysis of variance (ANOVA) (Holm-Sidak method).

## Results

### Haptoglobin and hemopexin inhibit vaso-occlusion

**Fig 1:**
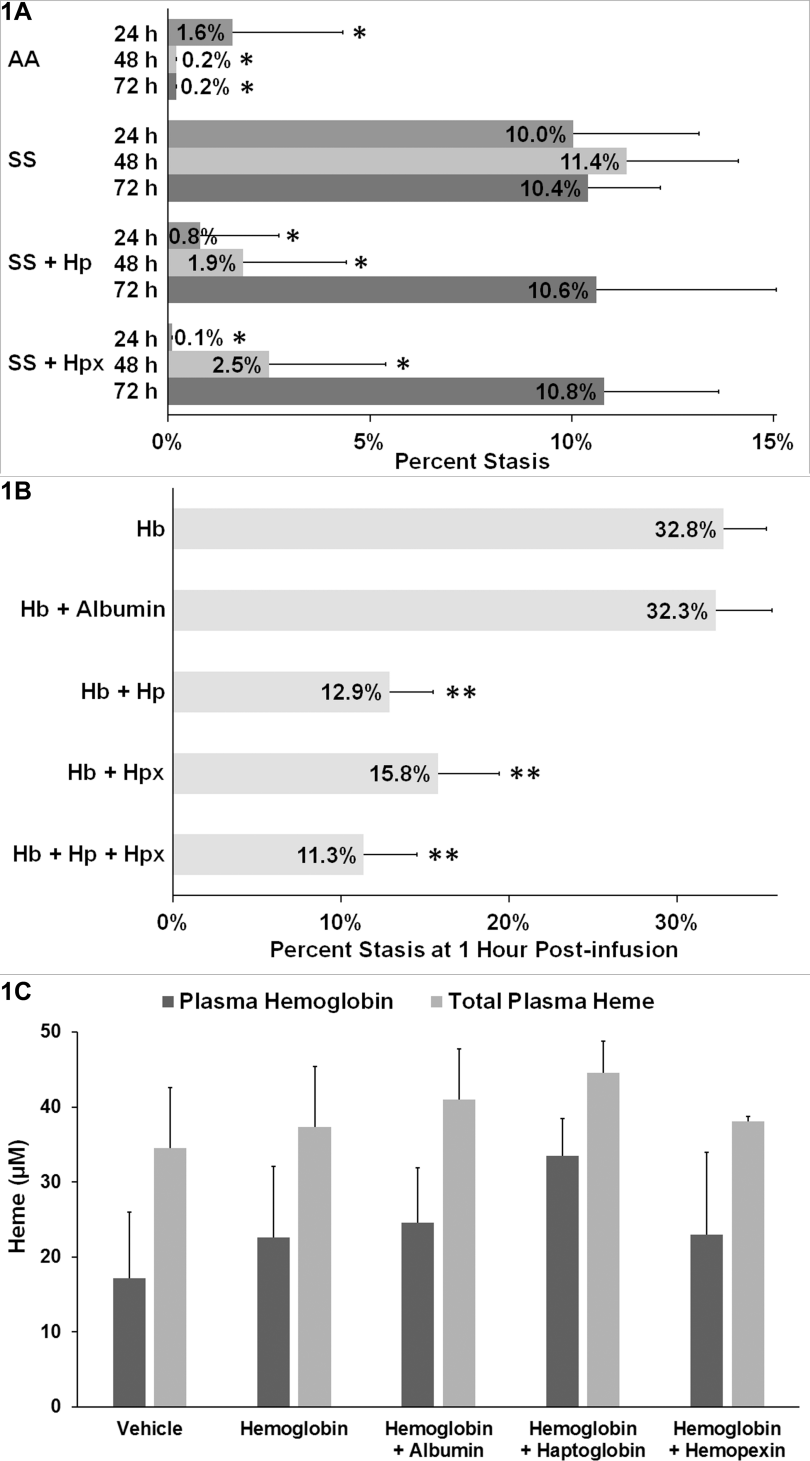
Haptoglobin and hemopexin inhibit hemoglobin-induced stasis in SS-mice. Dorsal skin-fold chambers were implanted onto Townes-SS mice (n = 4/group) and Townes-AA mice (n = 3/group) and 20–24 flowing venules were selected in each mouse at baseline (time 0). **(A)** SS and AA-mice were untreated or SS-mice were infused with haptoglobin (Hp, 1 μmol/kg) or hemopexin (Hpx, 1 μmol/kg) at baseline after selection of flowing venules. Microvascular stasis (% non-flowing venules) was measured in the same venules at 24, 48 and 72 hours. Bars represent means ± SD. *P < .05 versus SS (untreated). **(B)** SS-mice with implanted dorsal skin-fold chambers (n = 3/group) were infused with equimolar concentrations (1 μmol/kg) of hemoglobin (Hb), Hb + albumin, Hb + Hp, Hb + Hpx, or Hb + Hp + Hpx (0.5 μmol/kg each of Hp and Hpx). Microvascular stasis was measured 1 hour after infusion. Values are means ± SD. **P ≤ .01 versus Hb and Hb + albumin. **(C)** SS-mice without dorsal skin-fold chambers (n = 4/group) were infused with vehicle (saline), Hb, Hb + albumin, Hb + Hp, or Hb + Hpx at equimolar concentrations (1 μmol/kg). Total plasma heme and Hb levels were measured in venous plasma samples collected 1 hour after infusion. Bars are means ± SD.

We have previously shown that the hyperhemolytic Townes-SS mouse model develops spontaneous, unprovoked microvascular stasis (vaso-occlusion) in steady-state that can be inhibited by daily infusion of haptoglobin (Hp) or hemopexin (Hpx) [5]. In the current set of studies we questioned how long a single infusion of purified human Hp or Hpx would inhibit stasis in unchallenged SS-mice. After baseline selection of flowing venules in SS-mice, a single dose of Hp or Hpx (1μmol/kg) was infused at time zero and stasis was measured in the same venules 24, 48 and 72h after Hp or Hpx infusion **(Fig 1A)**. Control untreated AA-mice had little or no stasis, while untreated SS-mice had 10–11% stasis at 24, 48 and 72h. In contrast, SS-mice infused once with Hp or Hp at baseline had <1% stasis 24h after infusion, ~2% stasis at 48h, and 10–11% stasis at 72h. Thus a single infusion of Hp or Hpx inhibited stasis for 48h.

Infusion of exogenous hemoglobin into SS-mice can markedly increase stasis compared to the amount of spontaneous stasis [5]. We next examined the effectiveness of Hp and Hpx relative to albumin in SS-mice provoked with exogenous hemoglobin (Hb). Hb induces maximal stasis within 1h in SS-mice (data not shown). Therefore, we measured microvascular stasis one hour after infusion of Hb alone or with equimolar Hp, Hpx, or albumin (1μmol/kg). Sickle mice co-infused with Hb + Hp, Hb + Hpx or Hb + Hp + Hpx had less stasis 1h after infusion than mice infused with Hb + albumin or Hb **(Fig 1B)**. Surprisingly, Hp or Hpx inhibited Hb-induced stasis to a similar degree as the combination of Hp + Hpx.

To test the hypothesis that Hp and hemopexin lower plasma Hb and heme levels, we measured plasma Hb and heme levels in SS-mice (n = 4/group) 1h after infusion of vehicle (saline), Hb, Hb+albumin, Hb + Hp, or Hb + Hpx (1μmol/kg). There were no statistically significant differences in plasma Hb or total heme levels between the treatment groups at one hour after infusions **(Fig 1C)** suggesting that any effects of treatments on plasma Hb and heme levels were short-lived at the dose used. We estimate the amount of infused Hb-heme represented ~39% of the total plasma heme in circulation and would have been cleared from circulation in ~9.5 minutes **(S1 Calculation)**. Thus, plasma Hb and heme levels were not significantly different between the treatment groups one hour after infusion despite the marked differences in stasis.

### Haptoglobin and hemopexin inhibit markers of inflammation

**Fig 2:**
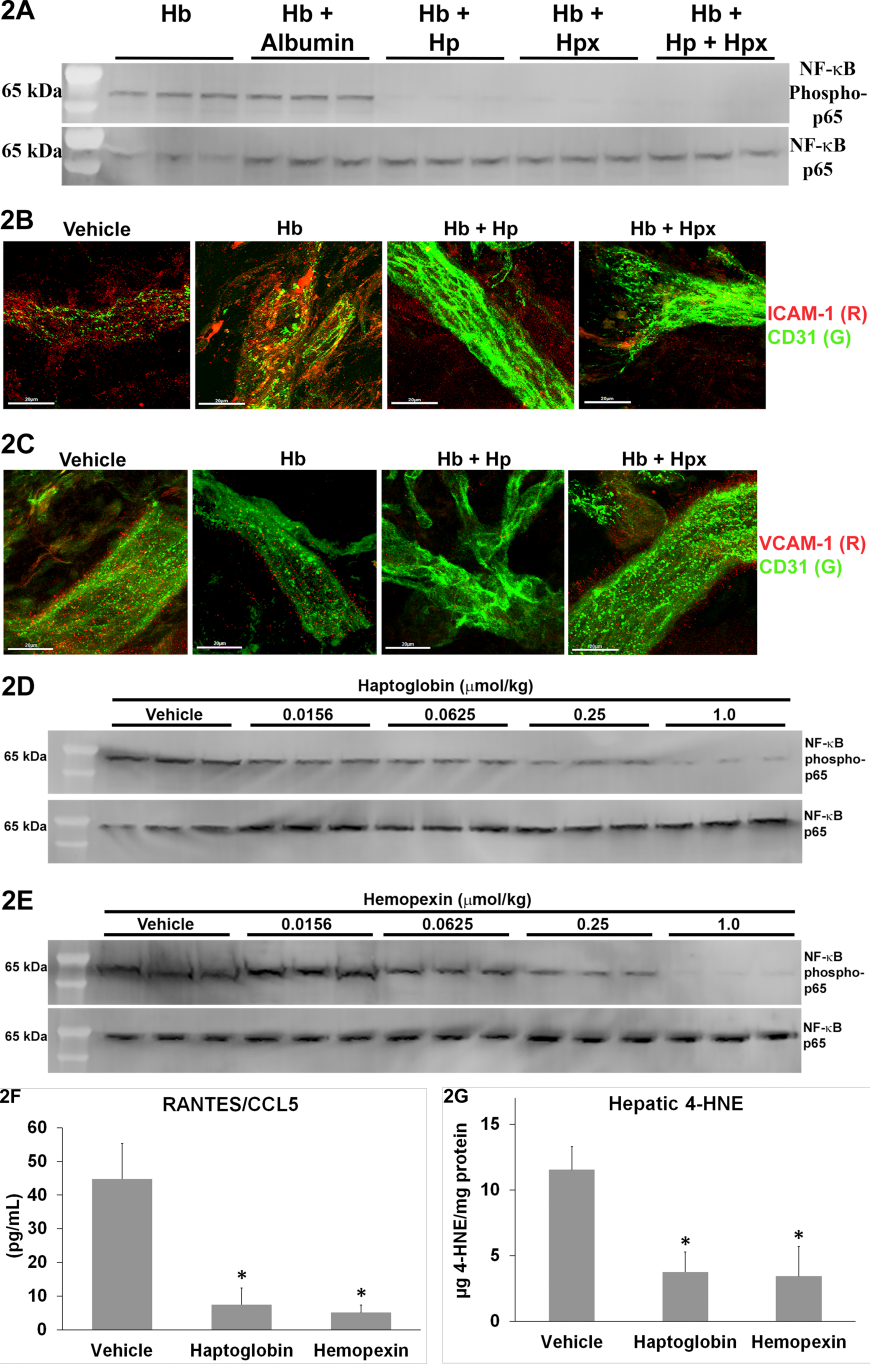
Haptoglobin and hemopexin inhibit inflammatory markers. **(A)** SS-mice (n = 3/group) were infused with equimolar concentrations (1 μmol/kg) of Hb, Hb + albumin, Hb + Hp, Hb + Hpx, or Hb + Hp + Hpx. Livers were removed and flash frozen 4 hours after infusion. NF-κB phospho- and total p65 expression was assessed in hepatic nuclear extracts by immunoblot. **(B and C)** ICAM-1 (red) or VCAM-1 (red) and CD31 (green) immunofluorescence in dorsal skin samples from SS-mice 4 hours after infusion of vehicle, Hb, Hb + Hp, or Hb + Hpx. **(D and E)** SS-mice (n = 3/group) were infused with vehicle or increasing doses (0.0156, 0.0625, 0.25 or 1.0 μmols/kg) of Hp or Hpx at baseline. Livers were removed and flash frozen 24 hours after infusion. NF-κB phospho- and total p65 expression was assessed in hepatic nuclear extracts by immunoblot. **(F)** RANTES/CCL5 levels in the plasma of SS-mice (n = 3/group) 24 hours after infusion of vehicle, Hp or Hpx (1 μmol/kg). Bars are means ± SD, *p < .01 versus vehicle). **(G)** 4-Hydroxynonenal (4-HNE) levels in liver microsomes of SS-mice (n = 3/group) 24 hours after infusion of vehicle, Hp or Hpx (1 μmol/kg). Bars are means ± SD, *p < .01 versus vehicle.

NF-κB is a major pro-inflammatory transcription factor. Nuclear expression of NF-κB phospho-p65 is a measure of NF-κB activation [42]. Compared to Hb alone or Hb + albumin infused animals, sickle mice co-infused with Hb + Hp, Hb + Hpx, or Hb + Hp + Hpx had markedly diminished hepatic NF-κB activation 4h after infusion as evidenced by nuclear NF-κB phospho-p65 levels **(Fig 2A)**. Expression of total NF-κB p65 was similar in all groups.

NF-κB activation promotes the transcription of pro-inflammatory adhesion molecules that are required for stasis in the dorsal skin-fold chamber model [5]. Immunofluorescence staining of VCAM-1 and ICAM-1 was decreased in the dorsal skin of SS-mice 4h after infusion of Hb+Hp and Hb+Hpx compared to SS-mice infused with vehicle or Hb **(Fig 2B and 2C)**.

Even after 24 hours nuclear expression of NF-κB phospho-p65 in the livers of SS-mice was inhibited by infusion of haptoglobin **(Fig 2D)** or hemopexin **(Fig 2E)** in a dose-responsive manner. Similar dose-responsive anti-inflammatory responses were seen in the kidneys (data not shown).

Pro-inflammatory cytokine RANTES (chemokine ligand 5 or CCL5), which recruits leukocytes to pro-inflammatory sites, was lower in the plasma of SS-mice 24 hours after infusion of Hp or Hpx compared to vehicle-treated SS-mice **(Fig 2F)**. However, there were no significant differences in plasma TNF-α, IL-10 and IFN-γ (data not shown). Hepatic 4-hydroxynonenol (4-HNE), a marker of oxidative stress, was significantly lower in the liver microsomes of SS-mice 24 hours after infusion of Hp or Hpx compared to vehicle-treated SS-mice **(Fig 2G)**.

### Haptoglobin and hemopexin induce HO-1 expression and activity

**Fig 3:**
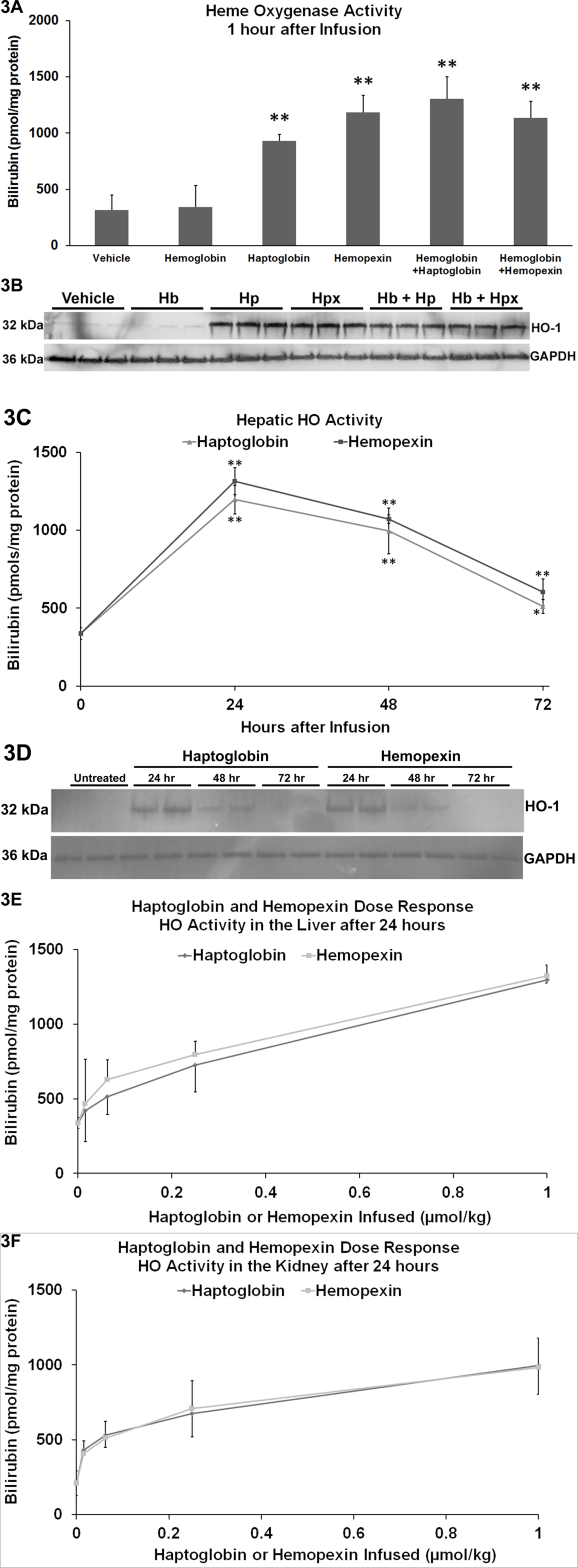
HO-1 is rapidly increased after haptoglobin and hemopexin infusion. **(A and B)** SS-mice (n = 3/group) were infused with vehicle or equimolar (1 μmol/kg) Hb, Hp, Hpx, Hb + Hp, or Hb + Hpx. Livers were removed and flash frozen 1 hour after infusion. Hepatic microsomes were used to assess heme oxygenase (HO) activity **(A)** via bilirubin production and protein expression **(B)** via immunoblot. Bars are means ± SD, **p < .01 versus vehicle or Hb. **(C and D)** SS-mice (n = 3/group) were untreated or infused with Hp or Hpx (1 μmol/kg) at baseline (time 0). Livers were removed and flash frozen 24, 48 or 72 hours after infusion. Hepatic microsomes were used to assess **(C)** HO activity and **(D)** HO-1 protein expression via immunoblot. Bars are means ± SD, *p < .05 and **p < .01 versus untreated SS-mice. **(E and F)** SS-mice (n = 3/group) were infused with vehicle or increasing doses (0.0156, 0.0625, 0.25 or 1.0 μmols/kg) of Hp or Hpx at baseline. Livers and kidneys were removed and flash frozen 24 hours after infusion. Hepatic **(E)** and kidney **(F)** microsomes were used to assess HO activity. Bars are means ± SD.

Since Hp-Hb and Hpx-heme can induce the anti-inflammatory enzyme HO-1 through a process that involves receptor-mediated endocytosis [43, 44], we examined hepatic HO-1 expression in response to Hb, Hp and Hpx in SS-mice. Hp and Hpx, but not Hb, increased hepatic HO activity 3 to 4-fold one hour after infusion compared to vehicle-treated SS-mice **(Fig 3A)**. Similarly, Hp and Hpx, but not Hb, markedly increased HO-1 protein expression on immunoblots one hour after infusion compared to vehicle **(Fig 3B)**. HO-1 induction was equally robust with or without co-infusion of exogenous Hb. Induction of hepatic HO-1 infused with Hb + albumin was not significantly different from Hb-infused mice (data not shown). In the dorsal skin where stasis was measured and the kidneys, HO-1 protein expression was increased 3 to 5-fold 1h after Hb + Hp or Hb + Hpx infusion versus vehicle **(S1 Fig)**. Hepatic HO activity **(Fig 3C)** and HO-1 protein **(Fig 3D)** remained elevated at 24 and 48 hours after Hp and Hpx infusion and dropped closer to baseline after 72 hours. The duration of enhanced HO activity in the liver induced by Hp and Hpx was similar to duration of stasis inhibition seen in Fig 1A.

In dose-response experiments, increasing amounts of Hp or Hpx from 0 to 1.0 μmol/kg were infused into SS-mice. Twenty-four hours after infusion, HO activity was measured in the liver **(Fig 3E)** and kidneys **(Fig 3F)**. HO activity increased dose responsively in both organs. Hp and Hpx had nearly identical effects on HO activity in both organs. Similar findings were seen on HO-1 immunoblots (data not shown).

**Fig 4:**
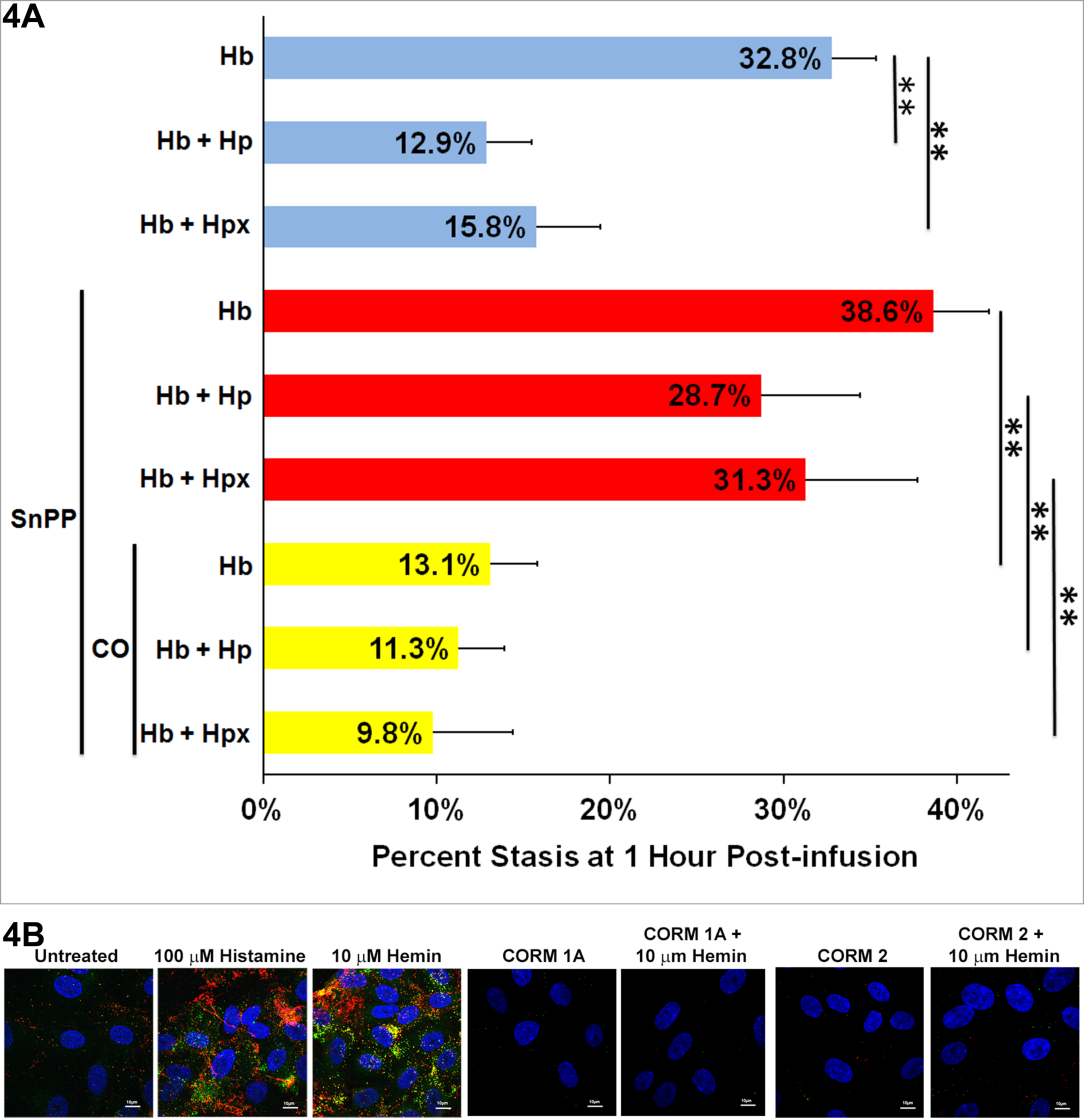
The HO-1 inhibitor tin protoporphyrin (SnPP) blocks the inhibition of stasis by haptoglobin and hemopexin and is reversed by carbon monoxide (CO). **(A)** Three groups of SS-mice (n = 9/group) with implanted dorsal skin-fold chambers were infused with equimolar (1 μmol/kg) Hb (n = 3), Hb + Hp (n = 3), or Hb + Hpx (n = 3). The first group (blue bars) had no pretreatments prior to infusion. The second group (red bars) was pretreated with the HO inhibitor SnPP (40 μmol/kg i.p. X 3 days) prior to infusion. The third group (yellow bars) was pretreated with SnPP and inhaled CO (250 ppm in air X 1h/day X 3 days) prior to infusion. Immediately prior to infusion, 20–24 flowing venules were selected in each mouse. Microvascular stasis was measured in the same venules 1 hour after infusion. Bars represent means ± SD. **P < .01. **(B)** Human umbilical vein endothelial cells (HUVEC) were incubated ± 80 μM CO-releasing molecule (CORM) 1A or CORM 2 for 30 minutes followed by treatment with 10 μM hemin for 30 minutes. HUVEC treated with 100 μM histamine served as a positive control for Weibel-Palade body P-selectin and VWF expression on the cell surface. Green and red fluorescence denote P-selectin and VWF expression, respectively, on the surface of HUVEC. The blue fluorescence denotes nuclei. Magnification is 60X. White bars in images represent 10 μm.

We have previously shown that induction of HO-1 expression or liver-directed HO-1 gene therapy inhibits stasis in sickle mice exposed to H/R [38, 45]. To determine if HO-1 was mediating the protective effects of Hp and Hpx, we pretreated SS-mice with the HO-1 inhibitor, tin protoporphyrin (SnPP, 40 μmol/kg/day i.p. X 3 d). SnPP inhibited hepatic HO activity in SS-mice infused with Hb + Hp or Hb + Hpx (data not shown). SnPP also reversed the inhibitory effects of Hp and Hpx on Hb-mediated stasis **(Fig 4A, blue bars -SnPP and red bars + SnPP)**. However, pretreatment of SS-mice with SnPP and inhaled CO, an HO reaction product, blocked the negative effects of SnPP **(Fig 4A, yellow bars).** CO also inhibited stasis in mice treated with SnPP + Hb without Hp or Hpx. Stasis was lower in all mice treated with SnPP + CO compared to mice treated with SnPP without CO. SnPP and CO had similar inhibitory effects on NF-kB activation in the liver (data not shown).

P-selectin is an important determinant of microvascular flow in sickle mice [46, 47]. In a clinical trial, blockade of P-selectin with a monoclonal antibody resulted in a significantly lower rate of pain crises in SCD patients [48]. P-selectin is intimately associated with von Willebrand factor (VWF) in endothelial cell Weibel-Palade bodies (WPB) and P-selectin anchors VWF to the vessel wall [49, 50]. We hypothesized that CO was inhibiting heme-mediated P-selectin and VWF expression on endothelium. P-selectin and VWF in WPBs can be rapidly mobilized to endothelial cell and vessel wall surfaces by heme-mediated TLR4 signaling [5] and triggers microvascular stasis in SS-mice. To test our hypothesis, human umbilical vein endothelial cells (HUVEC) were pretreated for 30 minutes with CO-releasing molecules (CORM) 1A or CORM 2 before adding heme to the media. In the absence of CORM, heme or the positive control histamine induced P-selectin (green) and VWF (red) onto the surface of HUVEC **(Fig 4B)**. Pretreatment of HUVEC with CORM 1A or CORM 2 blocked heme-induced expression of P-selectin and VWF on HUVEC cell surfaces. Thus, Hp and Hpx appears to inhibit stasis in SS-mice by inducing HO activity leading to more CO production, which inhibits WPB mobilization of P-selectin and VWF to the vessel wall.

### Haptoglobin and hemopexin also inhibit microvascular stasis induced by hypoxia-reoxygenation (H/R) and LPS

**Fig 5:**
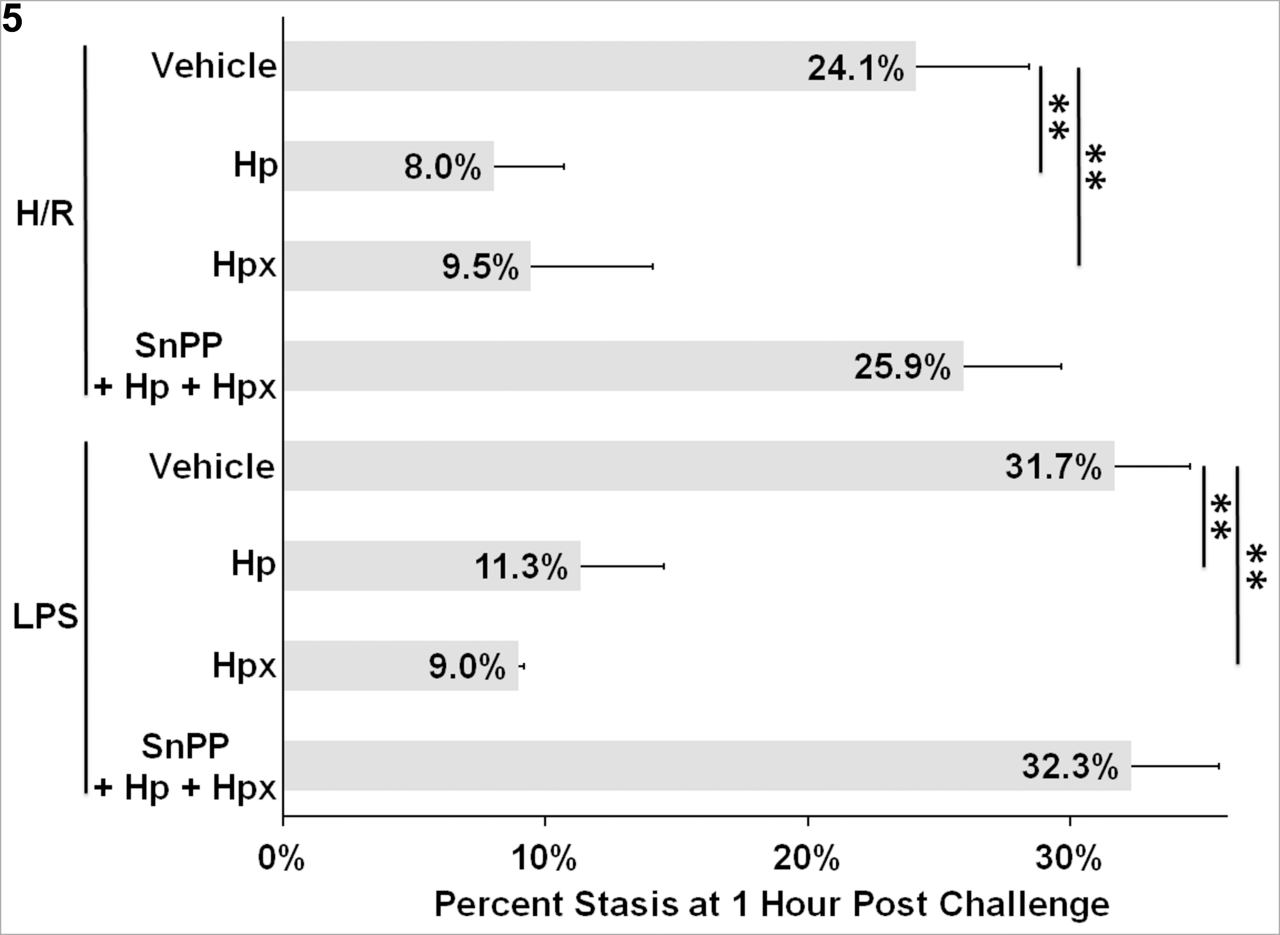
Haptoglobin and hemopexin inhibit stasis in SS-mice challenged with hypoxia-reoxygenation (H/R) and lipopolysaccharide (LPS). Dorsal skin-fold chambers were implanted onto SS-mice (n = 3/group) and 20–25 flowing venules were selected in each mouse. After venule selection, mice were infused with vehicle, Hp (1 μmol/kg), Hpx (1 μmol/kg), or Hp + Hpx (0.5 μmol/kg each of Hp and Hpx). The Hp + Hpx mice were pretreated with SnPP (40 μmols/kg i.p. X 3 days). One hour after infusion, mice were challenged with H/R (7% O_2_ for 1h followed by room air for 1h) or LPS (1 mg/kg, i.p.). Microvascular stasis was measured after H/R or one hour after LPS administration. Bars are means ± SD. **P < .01.

H/R and LPS can mobilize WPB P-selectin and VWF and trigger microvascular stasis in SS-mice. We tested whether Hp and Hpx could prevent stasis in response H/R and LPS. Hp and Hpx significantly inhibited stasis in response H/R or LPS **(Fig 5)**. Inhibition of HO activity with SnPP reversed the inhibition of stasis by Hp + Hpx.

## Discussion

Plasma Hp and Hpx levels are depleted in SCD mice and patients [21–24]. The current studies used the hyperhemolytic Townes-SS mouse model to examine the protective effects of Hp and Hpx supplementation and explore the role of HO-1 in that protection. SS-mice infused with Hp or Hpx had significantly less microvascular stasis after infusion regardless of whether they were unchallenged or challenged with Hb, H/R or LPS. These studies provide several new findings regarding Hp and Hpx in SS-mice. First, Hp and Hpx were equally effective in inhibiting stasis, suppressing NF-κB activation, and inducing HO-1. Second, inhibition of Hb-mediated effects by Hpx is consistent with the release of heme from metHb in the circulation of SS-mice as reported by this group previously [5]. Third, HO-1 protein and activity were rapidly increased within 1h of Hp or Hpx infusion and remained elevated for 48 to 72 hours after infusion paralleling the inhibition of stasis. The HO-1 response to Hp and Hpx in the liver and kidney was dose responsive. Fourth, the inhibition of microvascular stasis by Hp and Hpx was dependent on HO activity. The beneficial effects of Hp and Hpx were lost by inhibition of HO with SnPP, but could be restored by CO administration. Fifth, P-selectin and VWF expression, important determinants of microvascular flow in SCD, were blocked by pre-incubating HUVEC with CORMS.

The similar timing and effects of Hp and Hpx in SS-mice suggests there might be common pathways for Hb-Hp and heme-Hpx clearance and/or signaling in mice. Contrary to humans, murine Hp does not promote high-affinity binding to CD163 [51]. However, an alternative receptor for Hb-Hp in mice has not been identified. Human hepatocytes in primary culture express about 84,000 Hb-Hp receptors per cell [52]. Plasma clearance studies in rodents show a fast clearance (half-life a few minutes) of Hb-Hp and heme-Hpx from plasma [16, 51]. Heme-Hpx binds to a scavenger receptor, LDL receptor–related protein-1 (LRP1/CD91), which is expressed in many cell types, thus suggesting multiple tissue sites for heme-Hpx removal [12]. However, the bulk of heme-Hpx uptake by LRP1 occurs in the liver [22]. The similarity of the timing and effects of Hp and Hpx in SS-mice suggest that Hp and Hpx could potentially share common clearance and/or signaling pathways in mice that might differ from humans. Equimolar infusions of Hp or Hpx with Hb provided similar protection against stasis and HO-1 induction and had similar dose-response effects in the liver and kidneys suggesting not only a common clearance pathway in SS-mice, but also quantitative release of heme from Hb in the absence of Hp. In contrast, albumin did not induce HO-1 or inhibit stasis and NF-κB. Albumin can complex with heme *in vivo*, especially in haptoglobin-depleted states such as SCD [4, 53]. However, albumin has a much lower binding-affinity for heme (K_d_ = ~4 X 10^−5^ M) versus Hpx (K_d_ < 10^−13^ M) and thus heme-Hpx complexes predominate in sera when there is sufficient Hpx [11]. The lack of protection against stasis by albumin might not be surprising, as endogenous circulating serum albumin in mice, which is ~4 g/dL or ~600 μM, does not prevent heme-mediated TLR4 activation and stasis in SS-mice in response to heme infusions as low as 0.32 μmol/kg [5].

We initially hypothesized that Hp and Hpx might be protective in SS-mice through the removal of Hb and heme from the circulation. But Hb and heme remained highly present in circulation. Total Hb and heme levels in the plasma of SS-mice were not different between treatment groups 1h after any of the infusions. This suggests a rapid throughput of Hb and heme in the plasma of SS-mice. The Hb-heme clearance rate in SS-mice infused with 1μmol/kg Hb was estimated to be 9.5 minutes based on exhaled CO levels in SS-mice **(S1 Calculation)**. This calculation agrees with the rapid increase in HO-1 in the liver and skin seen 1h after of Hp/Hpx infusion, even in the absence of exogenous Hb. The inhibition of stasis in SS-mice challenged with H/R or LPS and the requirement for HO activity for this protection demonstrate that Hp and Hpx have anti-inflammatory properties linked to HO-1 induction.

In our study, infusion of Hb, which releases free heme in SS-mice, had little effect on hepatic HO-1 expression 1 to 4 hours after infusion. In a recent study by Graw et al [54], administration of storage-injured red blood cells (RBCs) in mice triggered *Hmox1* (HO-1) mRNA induction in liver, kidneys, and spleen and hepatic *Hmox1* mRNA was similarly increased by co-infusion of albumin, Hp, or Hpx. Important conclusions of this study seem to contradict our findings. However, there are several major differences between the two studies. First, they used a murine model of hemorrhagic shock and the current study used a murine model of SCD. These two models are likely to be physiologically dissimilar. Second, they administered fresh or stored RBCs. Splenic or hepatic phagocytosis of RBCs by tissue macrophages may activate different signaling pathways compared to free Hb. Third, they infused ~18 μmol Hb-heme/kg versus 1μmol of Hb-heme/kg in the current study. Fourth, we used equimolar Hb/heme, Hp and Hpx while they used much higher molar ratios. Thus, it may not be surprising the two studies found differences in HO-1 expression.

Previously in studies by Reddy Chintagari et al, we reported that Hp inhibited HO-1 induction in the kidneys of sickle mice relative to Hb alone [55]. This is the opposite of what was seen in the current studies. We believe these differences are most likely explained by the different sickle mouse models used in these studies. The previous study used the non-anemic NY1DD sickle mouse model which has a red blood cell half-life of ~7 days [35]. In those studies Hp appeared to decrease the delivery of Hb to the kidneys relative to Hb alone as seen with lower HO-1 expression in the proximal tubules of the kidneys of Hp-treated NY1DD mice. The current study used the hyperhemolytic Townes-SS model which has a RBC half-life of 2.5 days [35]. In this study Hp appeared to enhance Hb delivery to the kidneys relative to Hb alone as seen with higher HO-1 expression in kidneys of Hp-treated Townes-SS mice. These data suggest that kidney filtration in the Townes-SS mice may be partially compromised and allow Hp-Hb complexes access to Hp receptors on the proximal tubules of the cortex. A previous study by Shi et al supports our current observation that Hp supplementation increases HO-1 expression in the kidneys of Townes-SS mice [56].

HO-1 induction significantly inhibits markers of tissue inflammation and vaso-occlusion in SCD mice [45] and *HMOX1* gene polymorphisms that increase HO-1 expression are associated with reduced incidence of acute chest syndrome in SCD children [57]. In the current studies, inhibition of HO activity with SnPP abolished Hp and Hpx-mediated inhibition of stasis in SS-mice challenged with Hb, H/R or LPS demonstrating that rapid induction of HO-1 plays an important role in the protection seen after Hp and Hpx infusion. Previously, Hpx gene therapy targeted to the liver of SCD mice had similar protective effects that were dependent on HO-1 [24]. In previously published gene therapy studies by our laboratory, overexpression of a rat HO-1 transgene exclusively in the livers of mice inhibited vaso-occlusion in the skin of SCD mice, suggesting that expression of HO-1 in the liver has distal effects on the vasculature of the skin [38]. But even without these distal effects, Hp and Hpx induced HO-1 in the dorsal skin (S1 Fig) where stasis measurements were made. HO-1 is a potent inhibitor of NF-κB activation. NF-κB activation promotes the transcription of pro-inflammatory adhesion molecules that are required for stasis in the dorsal skin-fold chamber model. Immunofluorescence staining of VCAM-1 and ICAM-1 (Fig 2B and 2C) was decreased in the dorsal skin of SS-mice after infusion of Hb+Hp and Hb+Hpx, which is consistent with reduced inflammation in the skin. Unfortunately, we did not obtain enough nuclear extract protein from dorsal skin to examine NF-κB on immunoblots.

The current study provides mechanistic insights into how Hp and Hpx might be anti-inflammatory in SCD. Although SnPP might potentially have off-target effects on heme proteins other than HO, the data suggest the inhibitory effects of SnPP were mediated through inhibition of HO, as the HO reaction product CO, restored the protection lost in SnPP-treated mice. Thus, the central mechanism of Hp- and Hpx-mediated protection in SS-mice might be the upregulation of HO-1 and the production of the gasotransmitter CO. CO has previously been shown to inhibit microvascular stasis, NF-κB activation and VCAM-1 and ICAM-1 expression [45]. However, the inhibitory effects of Hp/Hpx on stasis occurred within 1h suggesting other, rapid, anti-inflammatory pathways might be affected by CO. Heme-induced expression of P-selectin and VWF on the vessel wall is a rapid trigger of microvascular stasis in SS-mice [5]. Antibody blockade of P-selection or VWF significantly inhibits stasis in SCD mice [5] and vaso-occlusive crises in SCD patients. We used cultured HUVEC to demonstrate that CO inhibited heme-mediated WPB mobilization of P-selectin and VWF to HUVEC surfaces. Thus, CO-mediated inhibition of WPB mobilization might mediate some of the anti-vaso-occlusive effects seen in Hp/Hpx-treated SS-mice.

**Fig 6:**
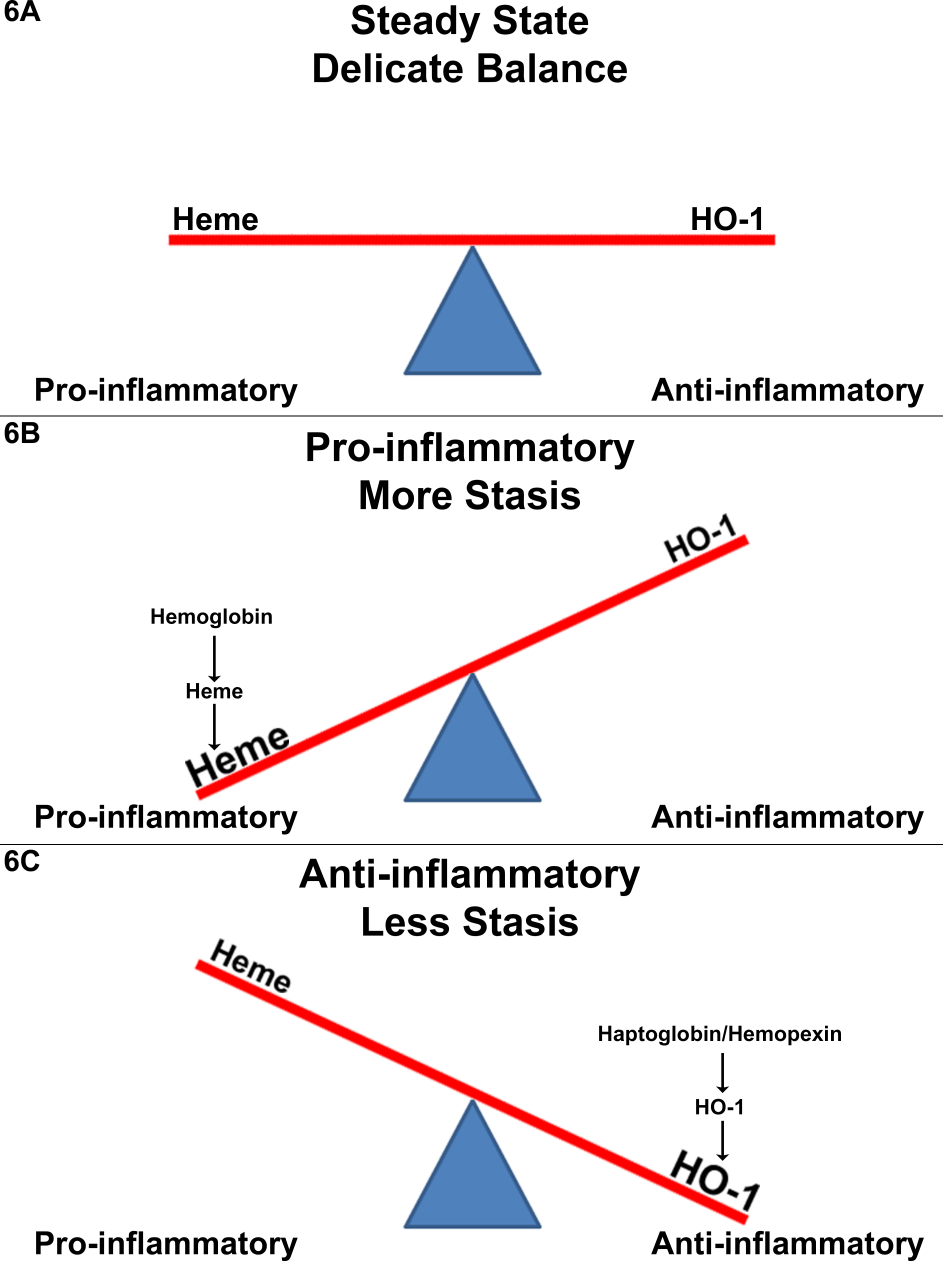
Tipping point: A delicate balance between pro-inflammatory and anti-inflammatory forces during steady-state sickle cell disease. Based on our data we propose a model of vaso-occlusion (stasis) in SCD where in steady-state pro-inflammatory heme and anti-inflammatory HO-1 are in a delicate balance **(A)**. Because of this delicate balance a relatively small increase in hemolysis or plasma hemoglobin/heme can tip the balance in favor of a pro-inflammatory state and more stasis **(B)**. And conversely, increasing HO-1 expression by administration of haptoglobin or hemopexin or other methods tips the balance in favor of an anti-inflammatory state and less stasis **(C)**.

Based on our data we propose a model of vaso-occlusion (stasis) in SCD where in steady state pro-inflammatory heme and anti-inflammatory HO-1 are in a delicate balance **(Fig 6A)**. Because of this delicate balance a relatively small increase in hemolysis or plasma hemoglobin/heme can tip the balance in favor of a pro-inflammatory state and more stasis **(Fig 6B)**. And conversely, increasing HO-1 expression by administration of haptoglobin or hemopexin or other methods tips the balance in favor of an anti-inflammatory state and less stasis **(Fig 6C)**. This model could help to explain why a vaso-occlusive crisis (VOC) can often come out of nowhere without warning. The model would imply that the rate of change in hemolysis may be more important than the absolute rate of hemolysis. An SCD patient with a lower rate of hemolysis can still have a VOC if the rate of hemolysis suddenly increases by a small amount because of the delicate balance between pro-inflammatory forces and the counterbalancing anti-inflammatory forces. It also implies that VOC might be prevented by increasing HO-1 pharmacologically thereby tipping the balance to anti-inflammatory protection. Although our model uses heme as the pro-inflammatory trigger for VOC, it is by no means the only trigger. Theoretically, any WPB agonist can induce VOC. The list of known WPB agonists is long and includes hypoxia, LPS, superoxide anion, thrombin, histamine, TNF-α, epinephrine, leukotrienes, acute shear stress, ATP/ADP, radiation, trauma, vasopressin, serotonin, fibrin, VEGF, complement C5a, ceramide, and sphingosine-1-phosphate. Unfortunately the list of known WPB antagonists is much shorter and includes N-acetylcysteine, CO, NO, and N-ethylmaleimide. We speculate that inducing the HO-1/CO axis with Hp/Hpx or with other pharmacologic agents will be globally effective against all of the WPB agonists. However caution is advised when using non-specific anti-oxidants, which may also suppress the Nrf2/HO-1 axis.

The anti-oxidant, anti-inflammatory, and anti-vaso-occlusive protections afforded by Hp/Hpx in SS-mice provide a rational basis for supplementation in Hp/Hpx-depleted SCD patients. Clinical studies of Hp/Hpx supplementation in SCD patients could potentially monitor tissue HO-1 expression longitudinally to determine optimal therapeutic dosing intervals. A recently published article showed an association between clinical improvement in SCD and repletion of Hp and Hpx by therapeutic plasma exchange using plasma replacement [58]. We speculate that Hp/Hpx supplementation may be beneficial in preventing or treating VOCs and acute chest syndrome in SCD patients as well as treating other hemolytic conditions.

## Supporting information

S1 Calculation Heme throughput in a 25g Townes-SS mouse. (DOCX)

S1 Fig Heme oxygenase-1 (HO-1) is rapidly increased in the dorsal skin after haptoglobin (Hp) and hemopexin (Hpx) infusion. Townes-SS-mice (n = 3/group) were infused with vehicle or equimolar (1 μmol/kg) hemoglobin (Hb), Hb + Hp, or Hb + Hpx. Dorsal skin was removed and flash frozen 1 hour after infusion. Skin microsomes were used to assess HO-1 protein expression via immunoblot. GAPDH was used as a loading control.
(DOCX)
