# Supplementary material for: Haptoglobin and hemopexin inhibit vaso-occlusion and inflammation in murine sickle cell disease: Role of heme oxygenase-1 induction
Source: PLoS One. 2018 Apr 25;13(4):e0196455. doi: 10.1371/journal.pone.0196455 (PMC5919001; doi:10.1371/journal.pone.0196455)
Supplement: S1 Calculation — (DOCX) [file pone.0196455.s001.docx]

**S1 Calculation for a 25g SS-mouse**

1. **Amount of heme, Hp and Hpx infused:** 1 µmol/kg X 25 g/mouse = **25 nmols**
2. **Total amount of heme in plasma:** ~40 nmols/ml X 1.6 ml plasma = ~**64 nmols**
3. **Infused heme, Hp or Hpx to circulating heme (%):** 25 nmols/64 nmols = ~**39%**
4. **Heme degradation rate:** (6.33nmol/h/g expired CO in 25g SS-mice) = ~**158 nmol/h**
5. **Clearance time of infused heme:** 25 nmols/158 nmols/60 min = **~9.5 minutes**

The plasma concentration of Hb infused (1 µmol heme/kg) is equivalent to 25 nmoles of heme in a 25 g mice (1 µmol heme/kg X 1000 nmol/µmol X 25 g X 1 kg/1000 g). The resting plasma Hb/heme concentration in SS-mice is ~40 µM or ~40 nmol/ml. A 25 g mouse has ~1.6 ml of plasma, thus the amount of heme in the plasma at any given moment is ~64 nmoles (40 nmol/ml X 1.6 ml). Therefore, the amount of Hb heme infused represents ~39% of the total heme in circulation (25 nmol infused/64 nmol in plasma). The plasma heme clearance rate can be estimated from the heme degradation rate. Exhaled CO quantitatively reflects the rate of heme catabolism [1]. The heme clearance rate in Townes-SS mice can be estimated by their expired CO, which is 6.33 nmol/h/g versus 1.0 nmol/h/g in control Townes-AA mice [2]. The metabolism of heme by heme oxygenase produces one mole of CO for each mole of heme degraded. Thus, in 25 g SS-mice, the heme clearance rate is ~158 nmol heme/h (6.33nmol/h/g X 1 hour X 25 g). So an infusion of 25 nmoles of Hb heme would be cleared in ~9.5 minutes (25 nmol/158 nmol/hour X 60 minutes/hour).

**Supporting References**

1. Coburn RF. Endogenous carbon monoxide production. N Engl J Med. 1970;282(4):207-9. Epub 1970/01/22. doi: 10.1056/nejm197001222820407. PubMed PMID: 4902834.

2. Belcher JD, Nath KA, Vercellotti GM. Vasculotoxic and pro-inflammatory effects of plasma heme: Cell signaling and cytoprotective responses. ISRN Oxidative Medicine. 2013;2013:Article ID 831596. Epub 2013/06/25. doi: 10.1155/2013/831596.
