# Supplementary material for: Haptoglobin and hemopexin inhibit vaso-occlusion and inflammation in murine sickle cell disease: Role of heme oxygenase-1 induction
Source: PLoS One. 2018 Apr 25;13(4):e0196455. doi: 10.1371/journal.pone.0196455 (PMC5919001; doi:10.1371/journal.pone.0196455)
Supplement: S1 Fig — Townes-SS-mice (n = 3/group) were infused with vehicle or equimolar (1 μmol/kg) hemoglobin (Hb), Hb + Hp, or Hb + Hpx. Dorsal skin was removed and flash frozen 1 hour after infusion. Skin microsomes were used to assess HO-1 protein expression via immunoblot. GAPDH was used as a loading control. (DOCX) [file pone.0196455.s002.docx]

**S1 Fig**

**
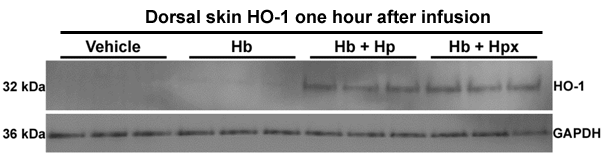
**

**Heme oxygenase-1 (HO-1) is rapidly increased in the dorsal skin after haptoglobin (Hp) and hemopexin (Hpx) infusion.** Townes-SS-mice (n=3/group) were infused with vehicle or equimolar (1 µmol/kg) hemoglobin (Hb), Hb + Hp, or Hb + Hpx. Dorsal skin was removed and flash frozen 1 hour after infusion. Skin microsomes were used to assess HO-1 protein expression via immunoblot. GAPDH was used as a loading control.
